# Prevalence and Associated Risk Factors of Helminth Infections in the Digestive Tract of Camels in Xinjiang, China

**DOI:** 10.3390/vetsci11100503

**Published:** 2024-10-14

**Authors:** Yi Zhang, Danchen Aaron Yang, Min Yang, Mengjie Pi, Yang Zhang, Zhanqiang Su

**Affiliations:** 1 Parasitology Laboratory, College of Veterinary, Xinjiang Agricultural University, Urumqi 830052, China; 120130015@xjau.edu.cn (Y.Z.); 320222772@xjau.edu.cn (M.Y.); 320242815@xjau.edu.cn (M.P.); 2 College of Veterinary Medicine, Nanjing Agricultural University, Nanjing 210095, China; d.a.yang@njau.edu.cn

**Keywords:** gastrointestinal helminth, detection, *Camelus bactrianus*, Xinjiang

## Abstract

**Simple Summary:**

Camels are one of the important economic animals in China; however, their health is often threatened by helminth. This study investigated the prevalence of helminth in camels by collecting 435 samples from five regions in Xinjiang using two methods. The results showed that 18.2% of the camels were infected with helminths, with the highest prevalence observed in Urumqi. Camels under three years old were found to be more susceptible. This study identified three major parasites (*Trichostrongylus* spp., *Chabertia ovina*, and *Haemonchus contortus*) in camels, and most camels were found to be simultaneously infected with multiple parasites. This research is crucial for formulating effective strategies to prevent and control camel parasites, and it contributes to improving the health and production efficiency of camels.

**Abstract:**

Camels, vital to economies in Asia, Africa, and the Arabian Peninsula, have been domesticated for over 4000 years. They thrive in arid regions like Xinjiang, China, but face challenges from internal and external parasites, particularly gastrointestinal parasites, which impact health, meat and milk quality, and production efficiency. This study investigates the prevalence of gastrointestinal helminth infections in camels from five major regions in Xinjiang. We collected 435 fresh fecal samples and used the saturated saline flotation method and McMaster’s method for detection. Molecular examination followed. The overall prevalence was 18.2% (95% confidence interval [CI]: 14.7–22.2%), with Urumqi showing the highest prevalence (29%, 95% CI: 23.4–35.1%) compared to other regions (odds ratio [OR]: 4.62, 95% CI: 2.63–8.41%). Younger camels (≤3 years old) were more likely infected by the parasites after adjusting for the region differences (OR: 10.53, 5.12–24.65%). However, we found no evidence that the prevalence was different between male and female camels. PCR analysis identified *Trichostrongylus* spp., *Chabertia ovina*, and *Haemonchus contortus* as predominant parasites, with observed co-infections indicating a complex parasitic landscape. The findings provide essential epidemiological data for effective parasite control strategies.

## 1. Introduction

Camels were domesticated around 4000 years ago for transportation, meat production, clothing manufacturing, and milk production [1,2]. Camel meat is of excellent quality, particularly in regions where other livestock struggle to thrive [1,2]. Camel milk, nutritionally superior to cattle milk, is rich in essential nutrients such as vitamins C and B, iron, and unsaturated fatty acids, and has a unique composition highly beneficial for human health [3]. Camels also produce milk for a longer duration than any other domesticated animals, providing a sustainable source of nutrition. Notably, in 2017, camels produced 2,852,213 tons of milk and 630,210 tons of meat [3]. In areas adapted to camels, they have served as valuable sources of income and transportation, especially before the invention of motorized transport and other economic developments [2]. In Xinjiang, China, camels are bred for their milk and are well suited to the arid and semi-arid rangelands of the region.

Internal and external parasites are common problems in camel breeding. Gastrointestinal parasites are the most prevalent pathogens in camels, causing nutritional and growth deficiencies and adversely affecting the quality of camel meat and milk [4,5,6,7,8,9,10]. Infected camels may suffer from diarrhea and other clinical symptoms, reducing production efficiency. Studies have identified the main parasites affecting camels in China, including *Ostertagia* spp., *Trichostrongylus* spp., *Haemonchus contortus*, *Nematodirus* spp., *Marshallagia* spp., *Trichuris* spp., *Chabertia ovina*, *Bunostomum* spp., *Strongyloides papillosus*, *Thysaniezia ovilla*, *Moniezia expansa*, *Dicrocoelium* spp., *Fasciola hepatica*, *Hasstilesia ovis*, *Eimeria* spp., *Giardia duodenalis*, *Neospora caninum*, *Theileria sinensis*, and *Habronematidae* [10,11,12,13,14]. These parasites not only impair the health of individual camels but also lower the overall productivity of camel herds, affecting milk and meat production. For instance, chronic infections can lead to decreased milk yield and quality, reducing the economic viability of camel milk production, especially in regions where camels are a primary source of dairy products [15]. With the rapid development of the camel industry in Xinjiang, the camel population has grown to 250,000. However, due to extensive grazing practices, camels are frequently infected with various gastrointestinal parasites, significantly affecting the industry’s development in Xinjiang. Although there has been an epidemiological investigation of digestive tract parasites of camels in Tianshan pastures [10], similar investigations in other regions of Xinjiang remain incomplete. Proper epidemiological studies and control strategies are essential to mitigate the impact of these parasites on camel populations. By understanding the prevalence, species distribution, and infection dynamics of camel gastrointestinal parasites, veterinarians and farmers can implement targeted prevention and treatment protocols [15]. This study aims to investigate the species and prevalence of camel gastrointestinal helminths in five main camel-producing areas in Xinjiang, providing epidemiological data to formulate prevention and control plans for camel parasites.

## 2. Materials and Methods

### 2.1. Study Areas

Xinjiang is home to 39 million sheep, 8.7 million cattle, 0.96 million horses, and 0.25 million camels. The five selected sites for this study are Urumqi (latitudes 42°45′ to 45°00′ north and longitudes 86°37′ to 88°58′ east), Aksu (latitudes 39°30′ to 41°27′ north and longitudes 79°39′ to 82° 20′ east), Altay (latitudes 47°27′ to 48°38′ north and longitudes 86°53′ to 88°37′ east), Hetian (latitudes 36°59′ to 37°14′ north and longitudes 79°50′ to 79°56′ east), and Ili (latitudes 43°50′ to 44°19′ north and longitudes 81°04′ to 81°29′ east). Urumqi is located in the central part of Xinjiang, at the heart of the Eurasian continent, and features a temperate continental climate. Aksu is situated in central Xinjiang and has a warm temperate continental arid climate. The Altay positioned in the northern of Xinjiang, is characterized by a typical temperate continental cold climate. Ili, lying in western Xinjiang, possesses both temperate continental and alpine climates. Hetian, located in the southern of Xinjiang, has an arid desert climate. These five zones are known for their high livestock and are also the main camel-producing areas. The number of camels in these regions totals 148,000, accounting for 59.2% of the total camel population in Xinjiang. The feeding modes of camels mainly include large-scale and backyard farms. Large-scale farms, with their extensive breeding operations, often employ modern technologies and management practices, particularly emphasizing disease control and prevention. In contrast, backyard farms, characterized by their limited scale, are typically run by herdsman families with minimal knowledge of disease management.

### 2.2. Sample Collection

The sampling procedures were carried out with the approval of the farm owner and in a manner that did not disrupt the farm’s regular operations. The sample collection started in September 2023 and finished in December 2023. The first author and three other trained technicians each visited a different large-scale camel farm in four distinct regions (excluding Urumqi) in September. While in Urumqi, the first author visited four backyard farms and one breeding center between September and December, with one farm and the breeding center visited in September and the others in subsequent months, one per month. Camels of all ages and sexes were included without discrimination. On each farm, the investigator spent up to 30 min in each pen to ensure thorough observation of the camels’ activities. Fresh, unoxidized fecal samples were collected and stored in separate containers immediately after the camels defecated, with one sample per container. Sample information was recorded directly on each container. To minimize the risk of cross-contamination, gloves were changed between fecal samples. After all samples were collected on the same day, they were transported (on ice) to the Parasitology Laboratory at the College of Veterinary Medicine, Xinjiang Agricultural University.

### 2.3. Morphological Examination

In this study, we employed a combination of the saturated salt water flotation method and the McMaster counting method to detect parasitic eggs in camel fecal samples. Initially, fecal samples were mixed with a saturated saline solution. The mixture was then strained to remove coarse particles, leaving behind a suspension enriched with eggs. This suspension was placed in a McMaster counting chamber. After allowing the chamber to rest for 5 min, enabling the eggs to float to the surface and settle evenly, the eggs were counted under a microscope.

### 2.4. Molecular Identification

Genome extraction of 79 fecal samples containing eggs was performed using the EasyPure^®^ Genomic DNA Kit (Transgen, Beijing, China). PCR amplification was then conducted on the DNA extracted from the eggs using nematode universal primers designed by Zhang [10]. The PCR was performed in a 50 μL reaction volume comprising 25 μL PrimeSTAR Max Premix (Takara, Shiga, Japan), 2 μL of extracted DNA template, 2 μL of each primer (10 μM), and sterile deionized water. The PCR conditions were as follows: an initial denaturation step at 98 °C for 5 min, followed by 30 cycles of denaturation at 98 °C for 10 s, annealing at 55 °C for 15 s, and extension at 72 °C for 15 s, with a final extension step at 72 °C for 10 min. The amplicons were analyzed using agarose gel electrophoresis on a 1% gel.

### 2.5. Statistical Analyses

The data analysis was followed by Yang et al. 2018 [16]. All analysis was performed in R (version 4.3.0) [17]. We first estimated the overall prevalence of animals found with parasite eggs with its 95% confidence interval (CI) being computed using the exact method (the 95% CIs were not calculated if the observed positive was zero). Before examining the relationships between the covariates (region, age and sex) and the prevalence, a one-way table was created to assess the counts of each level in a categorical variable. Levels with low counts were combined with adjacent levels if this was not biologically implausible, for example, age was combined into two categories, ≤3 years old and >3 years old. In the univariable analysis, two-way contingency tables were constructed to look at the association between each of the covariates and the outcome variable. A variable was recategorized by merging levels with low prevalences, for example, the region was merged into two levels—Urumqi and other regions. A chi-squared test was immediately performed to test whether there were significant differences in prevalences across the levels of a covariate. In addition, we performed bivariate analysis to study the associations between any pair of the covariates using two-way tables and chi-squared tests. Variables with *p*-value ≤ 0.2 (determined by the univariable analysis) were included in the multivariable logistic regression model, a backward elimination was implemented by dropping a variable with the highest *p*-value, until all variables were retained in the model with *p*-values < 0.05. A variable was forced in the model if confounding (removing the variable altering the coefficient or standard error of any other variable by ≥0.15) was required to control. Once the variable selection process was finished, two-way interactions were created for any pairs of the variables retained in the model.

## 3. Results

### 3.1. Descriptive Data Analysis

**Figure 1:**
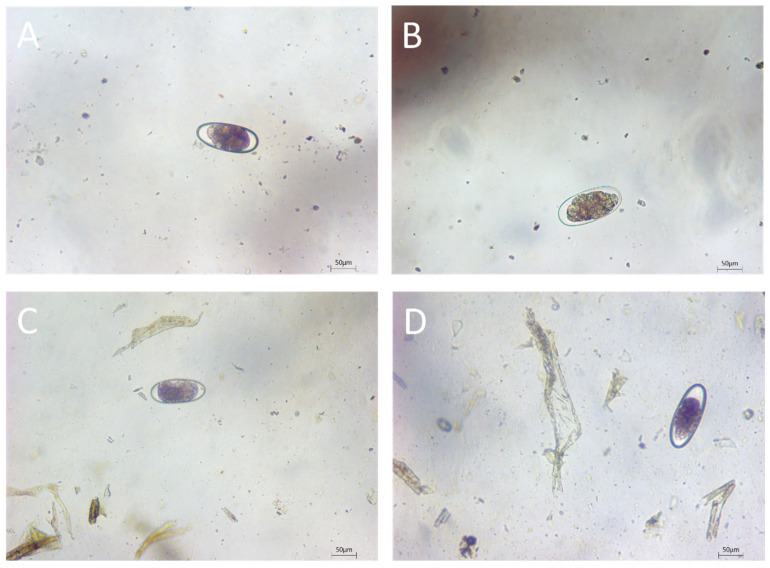
Morphological characteristics of nematode eggs in camel feces. (**A**–**D**) Trichostrongylidae eggs.

A total of 435 camel feces samples were collected, comprising 245 from Urumqi, 44 from Aksu, 37 from Altay, 33 from Hetian, and 76 from Ili. Of these, 79 samples were found to contain parasite eggs, yielding an estimated prevalence of 18.2% (95% CI: 14.7–22.1%). Figure 1 illustrates the morphological characteristics of nematode eggs in the sampled feces. Region-specific positive counts are summarized in Table 1, along with age- and sex-specific positive counts and estimated prevalences. Seventy-one of the positive samples were from Urumqi, while Aksu and Altay had two and six positive samples, respectively, with no positives found in Hetian or Ili. Table 2 shows the age and sex distributions across the regions. In Aksu, all sampled camels were older than three years, whereas all sampled camels in Altay were younger than three years. However, a chi-squared test indicated that age was not associated with the region when recategorized into a binary scale (Urumqi vs. non-Urumqi; *p*-value = 0.975). Regarding sex, Urumqi had a higher male-to-female ratio of approximately 1.5:1, such a pattern, however, was not observed in the other regions (*p*-value = 0.038 from a chi-squared test with four degrees of freedom). Furthermore, this study found a relationship between sex and age, with more males being over three years old (*p* < 0.0001). Particularly in Hetian and Ili, only male camels were found to be older than three years, while all younger sampled camels (≤3 years old) were females.

### 3.2. Infection Status in Different Regions, Age Groups, and Sex

The final logistic regression model included region and age group, and the age effect was not modified by region (Table 3). Sex was excluded from the model since it was neither significantly associated with the outcome nor considered a confounder. Higher prevalence was observed in camels in Urumqi compared to camels kept in other regions (odds ratio [OR]: 4.62, 95% CI: 2.63–8.41%). Younger camels (≤3 years old) were more likely infected by the parasites after adjusting for the region differences (OR: 10.53, 5.12–24.65%).

### 3.3. PCR Detection of Nematode Eggs Distribution and Co-Infection Patterns of Gastrointestinal Helminths in Camels

Among the 79 DNA samples, only 36 samples were amplified by PCR, and the target gene of the nematode was found. Out of a total of 36 infections, *Trichostrongylus* spp. was the most prevalent, accounting for 20 infections. *C. ovina* followed with 18 infections, while *H. contortus* was found in 11 cases. *Ostertagia* spp. and *Bunostomum* spp. were less common, with 3 and 2 infections, respectively. Co-infections were observed as well: *H. contortus* and *Trichostrongylus* spp. were co-present in four cases, and *H. contortus* and *C. ovina* in three cases. Other co-infections included *H. contortus* with *Ostertagia* spp. (one case), *Trichostrongylus* spp. with *C. ovina* (nine cases), and *Trichostrongylus* spp. with *Ostertagia* spp. (one case). Some co-infections involved three different parasites: *H. contortus*, *Trichostrongylus* spp., and *C. ovina* (one case); *H. contortus*, *C. ovina*, and *Ostertagia* spp. (one case); and *Trichostrongylus* spp., *C. ovina*, and *Ostertagia* spp. (one case) (Table 4).

In Urumqi, the most prevalent parasites are *Trichostrongylus* spp. and *C. ovina*, with 17 and 16 cases, respectively, while *H. contortus* is also significant with 6 cases. In Aksu and Altay, the total number of cases is considerably lower, with only a few occurrences of *H. contortus* and *Trichostrongylus* spp. Notably, *C. ovina* and *Ostertagia* spp. are absent in Aksu and rare in Altay. Combination infections are mostly observed in Urumqi, with *H. contortus* and *Trichostrongylus* spp. being the most common pair. Other combinations involving *H. contortus*, *C. ovina*, and *Trichostrongylus* spp. are also noted in Urumqi but are less frequent. This indicates a higher prevalence and diversity of parasitic infections in Urumqi compared to Aksu and Altay (Table 4).

*Trichostrongylus* spp. is the most frequently observed parasite among both sexes, affecting 11 males and 9 females. *C. ovina* is more common in males (12 cases) than females (6 cases). *H. contortus* also shows a higher prevalence in males (seven cases) compared to females (four cases). The infections of *Ostertagia* spp. and *Bunostomum* spp. are relatively rare, with *Ostertagia* spp. affecting two males and one female, and *Bunostomum* spp. was found only in males. Combination infections are predominantly observed in males, with notable cases of *H. contortus* and *Trichostrongylus* spp. (three cases), *H. contortus* and *C. ovina* (three cases), and *Trichostrongylus* spp. and *C. ovina* (six cases) (Table 4).

## 4. Discussion

Xinjiang is highly suitable for the development of animal husbandry due to its abundant grassland resources, suitable climatic conditions, and rich species resources [18,19,20]. Xinjiang is also one of the major production areas for camels [21]. According to reports, a camel breeding cooperative in Xinjiang generates an annual income exceeding CNY 2 million, and a single camel can bring in at least CNY 15,000 of income per year to local residents in Xinjiang. However, one of the biggest challenges to global camel production is parasitic diseases, which are major causes of impaired milk and production, decreases in performance, or even death [9,10]. The parasites infecting camels mainly include helminthic, arthropod, and protozoan [22]. In China, research on camel parasitic diseases mainly focuses on several types of parasites, including *Theileria*, *Cryptosporidium*, *Toxoplasma*, and *Cephalopina* [12,23,24,25]. Although the prevalence of camel gastrointestinal parasites is high, there is relatively little research on this topic.

The present study examined 435 camel feces samples to illustrate the prevalence of intestinal parasites among camels in five areas of Xinjiang, and the results showed that 79 samples were infected with intestinal parasites. A survey on the infection of digestive tract parasites in camels in some regions of Xinjiang from 2018 to 2019 showed that the prevalence was 31.4% (38/121) [26]. Research on gastrointestinal parasites in camels in the Tianshan Mountains pastoral area from 2016 to 2018 showed that the prevalence reaches 100% [10]. However, due to the limited sample size, a direct comparison for prevalence is not possible. Further longitudinal studies with larger sample sizes are required to monitor the prevalence of parasitic diseases over the years.

Comparing the prevalences across different areas, we found that Urumqi had the highest prevalence at 29%. Conversely, Aksu had a notably lower prevalence of 4.5%, and the prevalence of Altay was 16.2%. No infections were detected in Hetian or Ili. This high prevalence found in Urumqi was possibly due to the camel-rearing method in which camels kept on backyard farms were more likely to be infected by gastrointestinal parasites [27]. However, other factors, e.g., climatic differences could also contribute to the difference in prevalence. Urumqi lies in the northern part, which is wetter and colder compared to Aksu and Hetian. However, both hypotheses require further validation since the farm type effect is confounded by the regional/climate effect in this study.

In our study, camels aged ≤3 years showed a higher prevalence of 28% compared to older camels (OR: 10.53, 5.12–24.65). However, inconsistent findings were reported in the previous studies [2,28,29,30,31]. Some studies suggested that as the age of the camels increases, their exposure to parasites increases, resulting in higher prevalences [2,29,30]. Results from our study indicate that in addition to considering the exposure of camels to parasites, the factor of camels’ own resistance should also be considered.

The digestive tract of camels is rich in parasite species, with at least over 50 different types, and these parasites often exhibit a mix of parasitism [2,9,31]. In this study, PCR analysis of 79 DNA samples revealed that 36 contained target nematode genes, confirming the presence of *Trichostrongylus* spp., *H. contortus*, and *C. ovina* as the most common parasites. Twenty-one samples were detected with mixed infections of two pathogens, and three samples with mixed infections of three pathogens. Generally, the most common digestive tract parasites of camels are *Trichostrongylus* spp. and *H. contortus* [9,10]; however, this study found that the prevalence of *C. ovina* was relatively high. Through PCR, we also detected *Ostertagia* spp. and *Bunostomum* spp., but the prevalences were relatively low. Zhang’s research on camel digestive tract parasites in the Tianshan Mountains pastoral area identified a total of 15 species of parasites [10], while this study used PCR technology to confirm that there are 5 species of digestive tract parasites capable of infecting camels in the sampled areas. Therefore, further research should be conducted on this basis to gain a deeper understanding of the specific species of digestive tract parasites in Xinjiang camels.

Camels, as important economic animals in Xinjiang, have relatively little research conducted on their digestive tract parasites. The results of this study indicate that the prevalence of digestive tract parasites in Xinjiang camels is relatively high, and further systematic epidemiological investigations are still needed, as well as the development of comprehensive prevention and control measures.

## Figures and Tables

**Table 1:** Prevalence of gastrointestinal helminths found in 435 camels in Xinjiang.

Variable		Positives	Total	Prevalence (%)	95% Confidence Interval	Χ^2^ (DF) ^1^	*p*-Value
Region						44.172 (1)	<0.0001
Urumqi	Urumqi	71	245	29	23.4–35.1		
Other regions	Aksu	2	44	4.5	0.6–15.5		
	Altay	6	37	16.2	6.2–32		
	Hetian	0	33	0	NA		
	Ili	0	76	0	NA		
	Sub-total ^2^	8	190	4.2	1.8–8.1		
Age						26.484 (1)	<0.0001
≤3 years old		59	211	28	22–24.5		
>3 years old		20	224	8.9	5.5–13.5		
Sex						2.4454 (1)	0.1179
Female		40	186	21.5	15.8–28.1		
Male		39	249	15.7	11.4–20.8		
Total		79	435	18.2	14.7–22.2		

^1^: Indicated by the degree of freedom (DF), the chi-squared test for region is performed based on a 2-by-2 table where the region has two levels—Urumqi and other regions. ^2^: Sub-total includes the regions other than Urumqi.

**Table 2:** A matrix that describes the sex and age distributions of camels in the study areas in Xinjiang. Values in bold present the age and sex distributions in Urumqi and other regions as well as the age-specified female-to-male ratios.

Region/Sex		Age						Sex	
		≤3 Years Old			>3 Years Old			Female-Total	Male-Total
		Female	Male	Age-Total	Female	Male	Age-Total		
Urumqi	Urumqi	60	59	119	30	96	126	90	155
Other regions	Sub-total ^1^	72	20	92	24	74	98	96	94
	Aksu	0	0	0	24	20	44	24	20
	Altay	17	20	37	0	0	0	17	20
	Hetian	14	0	14	0	19	19	14	19
	Ili	41	0	41	0	35	35	41	35
								186	249
Total		132	79	211	54	170	224	435	

^1^: Sub-total includes the regions other than Urumqi.

**Table 3:** Effects of region and age on the gastrointestinal helminths infection.

Variable		Odds Ratio	95% Confidence Interval	*p*-Value
Region	Urumqi	4.62	2.63–8.41	<0.0001
	Other regions	ref		
Age	≤3 years old	10.53	5.12–24.65	<0.0001
	>3 years old	ref		

**Table 4:** Results for nematode infections in camel feces by region and gender.

	Infection Number	Urumqi	Aksu	Altay	Male	Female
*H. contortus*	11	6	1	4	7	4
*Trichostrongylus* spp.	20	17	1	2	11	9
*C. ovina*	18	16	0	2	12	6
*Ostertagia* spp	3	3	0	0	2	1
*Bunostomum* spp.	2	2	0	0	2	0
*H. contortus* and *Trichostrongylus* spp.	4	4	0	0	3	1
*H. contortus* and *C. ovina*	3	2	0	1	3	0
*H. contortus* and *Ostertagia* spp.	1	1	0	0	1	0
*Trichostrongylus* spp. and *C. ovina*	9	8	0	1	6	3
*Trichostrongylus* spp. and *Ostertagia* spp.	1	1	0	0	1	0
*Trichostrongylus* spp. and *Bunostomum* spp.	1	1	0	0	1	0
*C. ovina* and *Ostertagia* spp.	2	2	0	0	2	0
*H. contortus*, *Trichostrongylus* spp. and *C. ovina*	1	1	0	0	1	0
*H. contortus*, *C. ovina*, and *Ostertagia* spp.	1	1	0	0	1	0
*Trichostrongylus* spp., *C. ovina*, and *Ostertagia* spp.	1	1	0	0	1	0
Total	36	28	2	6	20	16

## Data Availability

Data are contained within this article, and further inquiries can be directed to the corresponding authors.
